# Investigating the Sharing of *Staphylococcus* spp. Between Dogs and Their Owners: A Comparative Study from Two Italian Veterinary Teaching Hospitals

**DOI:** 10.3390/pathogens15040356

**Published:** 2026-03-27

**Authors:** Francesca Paola Nocera, Patrizia Robino, Rossana Schena, Stefano Cavalli, Alessandro Bellato, Ilaria Prandi, Davide Mancusi, Annunziata Romano, Sinem Arslan, Giulia Iamone, Matteo Olimpo, Gerardo Fatone, Luisa De Martino, Patrizia Nebbia

**Affiliations:** 1 Department of Veterinary Medicine and Animal Production, University of Naples Federico II, 80137 Naples, Italy; francescapaola.nocera@unina.it (F.P.N.); rossana.schena@unina.it (R.S.); stefano.cavalli@unina.it (S.C.); annunziata.romano@unina.it (A.R.); sinem.arslan@unina.it (S.A.); gerardo.fatone@unina.it (G.F.); 2 Department of Veterinary Sciences, University of Turin, 10095 Turin, Italy; patrizia.robino@unito.it (P.R.); alessandro.bellato@unito.it (A.B.); ilaria.prandi@unito.it (I.P.); davide.mancusi@unito.it (D.M.); giulia.iamone@unito.it (G.I.); matteo.olimpo@unito.it (M.O.); patrizia.nebbia@unito.it (P.N.); 3 Task Force on Microbiome Studies, University of Naples Federico II, 80137 Naples, Italy

**Keywords:** *Staphylococcus* spp., human–dog microbial sharing, nasal colonization, antimicrobial resistance, One Health

## Abstract

Animal health is a key pillar of the One Health framework, which emphasizes the interconnectedness of humans, animals, and the environment. *Staphylococcus* spp., common commensals of skin and mucosa, are clinically important due to their virulence factors and increasing antimicrobial resistance. This cross-sectional study aimed to isolate and characterize *Staphylococcus* spp. from dogs and their owners and to assess correlations within their nasal microbiota. Nasal swabs were collected from at least 100 dog–owner pairs at two Veterinary Teaching Hospitals located in Northern (Turin Province) and Southern (Naples Province) Italy. In both study areas, *S. pseudintermedius* was the most common species in dogs. Among owners, *S. epidermidis* was predominant in Naples, while *S. epidermidis* and *S. aureus* were most frequent in Turin. A subset of 54 dog–owner pairs sharing the same *Staphylococcus* species (42 from Turin and 12 from Naples; in total 108 isolates) was included in this analysis, with a focus on antimicrobial patterns. *S. aureus* was the species most frequently shared between dogs and owners, followed by *S. epidermidis*, with no significant differences between the two sites. In particular, methicillin resistance (phenotypically inferred) was detected in 16.7% of isolates in Turin (19.0% in dogs; 14.3% in owners) and 41.7% of isolates in Naples (33.3% in dogs; 50.0% in owners). Multidrug resistance was detected in 34.3% of paired isolates overall, with a higher prevalence in Naples (58.3%) compared to Turin (27.4%). No significant association emerged between biofilm production and multidrug resistance (MDR). Overall, these findings suggest possible species sharing between dogs and owners, while biofilm formation did not predict MDR.

## 1. Introduction

Animal health is one of the pillars on which the One Health principle is based. The relationship between bacterial samples from dogs and their owners has become an increasingly important and widely discussed area of research in both microbiology and public health. As the recognition of the interconnectedness between human and animal health continues to grow, understanding how bacterial species, including pathogens, are shared between pets and their owners has significant implications for zoonotic disease transmission, antimicrobial resistance, and overall health management.

Recent studies support this perspective. For example, a 2024–2025 investigation identified a significant overlap in the gut microbiota of cohabiting human–dog pairs; specifically, 11 amplicon sequence variants (ASVs) were shared within these pairs, suggesting potential microbial exchange or similarity associated with cohabitation [1]. Similarly, new evidence shows that the ocular surface microbiomes of dogs and their owners exhibit increased similarity in households where they live together and that factors such as the size of the dog and the presence of other pets influence this sharing [2].

Moreover, the risk posed by antimicrobial-resistant bacteria shared between pets and humans has been underlined by genomic studies. A 2024 study using core genome multilocus sequence typing (cgMLST) documented cases of shared multidrug-resistant bacteria (e.g., resistant *E. coli* and vancomycin-resistant *E. faecium*) between dogs (and cats) and humans living in the same household, suggesting possible transmission or shared sources [3]. In a related manner, a 2024 study on dogs from diverse lifestyles (pet, rural, and stray) investigated their fecal microbiota and resistomes, reinforcing how environmental factors and lifestyle can shape the microbial and resistance profiles in dogs, with potential repercussions for health [4].

These findings align with the One Health approach by emphasizing the interconnectedness of humans, animals, and their environment. The evidence suggests that microbial sharing between pets and owners may have potential implications for public health. As this field of research evolves, further investigation is warranted to explore how these interactions might impact the prevention and control of infectious diseases that affect both species [5,6]. With the increasing number of companion animals in households, further study of these dynamics may provide valuable insights for strategies aimed at addressing emerging health threats in both animal and human populations.

*Staphylococcus* species are among the most common bacterial colonizers of both humans and companion animals, with prevalence on the skin, nasal mucosa, and other mucosal surfaces [7,8,9]. While many staphylococcal species function as commensals within the normal microbiota, several have the capacity to act as opportunistic pathogens, giving rise to infections when host defenses are compromised or microbial equilibrium is disrupted. In recent years, increasing attention has focused on the role of *Staphylococcus aureus*, *Staphylococcus pseudintermedius*, and related coagulase-negative staphylococci in the context of human–animal interactions, particularly within households where close contact facilitates bacterial sharing [10,11].

The emergence and dissemination of antimicrobial-resistant *Staphylococcus* strains including methicillin-resistant *S. aureus* (MRSA), methicillin-resistant *S. pseudintermedius* (MRSP), and multidrug-resistant coagulase-negative species represent a major challenge for both human and veterinary medicine. These bacteria frequently display resistance to multiple classes of commonly used antimicrobial agents, such as penicillins, cephalosporins, macrolides, lincosamides, and tetracyclines, limiting therapeutic options and increasing the complexity of clinical management. Recent studies in companion animals have documented high levels of multidrug resistance in both clinical and commensal isolates, with resistance profiles often involving several antibiotic classes simultaneously [7,12]. Additional work has confirmed that resistance to β-lactams, macrolides, and tetracyclines is widespread among *Staphylococcus* spp. that colonize dogs and cats, including healthy carriers [12], reflecting a broad and evolving resistance landscape.

Within the One Health framework, understanding the ecological and epidemiological dynamics of *Staphylococcus* spp. shared between humans and companion animals has become particularly relevant. Close physical contact between pets and their owners may facilitate cross-species transmission of both susceptible and resistant strains, contributing to the circulation of antimicrobial-resistant bacteria within households and potentially beyond [7,11]. Evidence of clonal relatedness between isolates from dogs and their owners reinforces the potential for interspecies bacterial exchange, particularly in settings characterized by frequent or prolonged human–animal interactions. These observations highlight the importance of integrated surveillance and coordinated antimicrobial stewardship strategies designed to protect the health of both humans and animals [13].

The aim of this study was to identify *Staphylococcus* species obtained from the nasal swabs of dogs and their owners at two Veterinary Teaching Hospitals in Italy, one located in Southern Italy (University of Naples) and the other in Northern Italy (University of Turin). Additionally, the antimicrobial resistance profiles of the isolated strains were analyzed. Special attention was given to cases in which the same *Staphylococcus* species was found in both the dog and its owner.

## 2. Materials and Methods

### 2.1. Ethical Statement

This study was approved by the Veterinary Service Center of the University of Naples Federico II (certificate number PG/2023/0120379) and by the Bioethics Committee of the University of Turin (certificate number 0251347), in compliance with Italian Legislative Decree 26/2014, Article 2, implementing Directive 2010/63/EU.

Nasal swab samples were collected from client-owned dogs undergoing routine surgical procedures at two Italian Veterinary Teaching Hospitals (University of Naples Federico II and University of Turin). Sample collection was performed by licensed veterinarians prior to surgery as part of standard clinical handling, causing minimal discomfort and no additional risk to the animals.

In accordance with the Declaration of Helsinki (1975, revised in 2013), nasal swab samples from dog owners were self-collected following written instructions provided by study personnel at the participating Italian universities and under supervision. No invasive procedures were performed, and sample collection did not involve any additional risk to the participants beyond routine personal hygiene practices.

Written informed consent was obtained from all participants prior to their inclusion in the study.

### 2.2. Study Design

A cross-sectional study involving dogs and their respective owners was performed at two Veterinary Teaching Hospitals (VTHs): VTH1 (University of Naples Federico II) located in Southern Italy and VTH2, University of Turin, located in Northern Italy. Subjects were enrolled at the time of the dogs’ admission for surgery. To ensure a clear geographical definition of the study populations, only dog–owner pairs residing in the same province as the recruiting hospital were eligible (Province of Naples for VTH1; Province of Turin for VTH2). Thus, the two study populations were defined at the provincial level, while enrollment was conducted at the corresponding referral hospitals. Upon arrival at the hospital and prior to sample collection, dog owners were provided with a written informed consent form. This included a brief questionnaire explaining the aim of the study, the procedures involved, and assurances regarding privacy and voluntary participation. After consent was obtained, paired nasal swabs were collected from each owner and their dog while the dog was under anesthesia and before the surgical procedure. Performing nasal swab sampling under these conditions minimized discomfort for the animals and facilitated the workflow of the veterinarians involved. The owners collected their own nasal swabs following the instructions provided. Participants who requested feedback received a summary of their individual results, including details of the *Staphylococcus* species identified in both the human and canine samples, as well as their antimicrobial resistance profiles.

### 2.3. Dog Owner Feedback Through Questionnaires

Dog–owner pairs were eligible for enrollment if they met the following criteria: (i) the dog’s admission to VTH1 or VTH2 during the study period and scheduling for elective or non-emergency surgical procedures; (ii) dogs of any breed or sex and aged ≥ 4 months; (iii) owner-reported residence within the Province of Naples (for VTH1) or within the Province of Turin (for VTH2); and (iv) provision of written informed consent and completion of a standardized questionnaire by the owner.

Pet owners were interviewed regarding their pet care practices and their relationship with their dogs. Specifically, the questionnaire was designed to collect information on the dog–owner relationship and close and prolonged contact behaviors with dogs that may facilitate the sharing of staphylococcal species and the dissemination of antimicrobial resistance determinants. The survey consisted of nine questions, each structured as multiple-choice items (2–4 response options), with the possibility of providing additional comments. The questions addressed the dog’s lifestyle and medical history, including any recent or previous antimicrobial treatments. A corresponding question regarding antimicrobial use was also included for the owners.

### 2.4. Sampling

This study was conducted between January 2024 and April 2025 at two sites: VTH1, Veterinary Teaching Hospital, University of Naples Federico II (Province of Naples, Campania Region, Southern Italy), and VTH2, Department of Veterinary Sciences, University of Turin (Province of Turin, Piedmont Region, Northern Italy). Sampling was performed on 120 dog–owner pairs at VTH1 and 160 dog–owner pairs at VTH2. Specimens consisted exclusively of non-invasive nasal swabs collected from dogs in the preoperative phase, primarily prior to orthopedic surgery. Nasal swabs were obtained from dogs while under sedation to minimize stress. Additionally, nasal swabs were collected from the dogs’ owners, who voluntarily agreed to participate in the study and performed the sampling themselves following the provided instructions. For each subject (both dog and owner), a single swab was inserted approximately 2–3 cm into each nostril, rotated 360° several times, and then placed in Stuart W/O CH transport medium (Aptaca S.p.A., Asti, Italy). All specimens were delivered to the respective microbiology laboratories for bacteriological analysis within a maximum of 2 h after collection.

### 2.5. Staphylococcus spp. Isolation and Identification

All samples were cultured on selective and differential agar media to isolate *Staphylococcus* spp. Mannitol salt agar (MSA) and Chromatic *Staph aureus* plates (Liofilchem srl, Teramo, Italy) were used and incubated aerobically at 37 °C for 24–48 h. Subsequently, the identification of the isolated strains was carried out by Matrix-Assisted Laser Desorption Ionization Time-of-Flight Mass Spectrometry (MALDI-TOF MS) analysis (Bruker Daltonics, Bremen, Germany). The obtained spectra were compared with those in the Biotyper database, with a log(score) ≥ 2.0 being considered valid. A bacterial test standard (BTS) (Bruker Daltonics, Bremen, Germany) was used as a calibrator for quality control. Quality control was ensured using the reference strains *S. pseudintermedius* (ATCC 49444, TUCC 0347) and *S. aureus* (ATCC 33591, TUCC 01025). Pure isolated colonies were stored in tryptone soy broth with 15% glycerol at −80 °C for subsequent analyses.

### 2.6. Antimicrobial Susceptibility Profiles

Antimicrobial susceptibility testing for *Staphylococcus* spp. was conducted by the Disk Diffusion Test (DDT) according to the European Committee on Antimicrobial Susceptibility Testing [14]. The analysis was performed exclusively on staphylococcal isolates belonging to the same species recovered from both members of each dog–owner pair. This targeted approach was adopted to focus the investigation on phenotypic indicators of sharing within households, allowing for a direct comparison of resistance profiles in isolates showing species-level concordance.

The antimicrobial agents tested were grouped according to their categories [14,15] as follows: penicillins (penicillin, P, 10 IU); β-lactams for detection of meticillin resistance, including oxacillin (OX, 1 μg) and cefoxitin (FOX, 30 μg); macrolides and lincosamides, represented by erythromycin (E, 15 μg) and clindamycin (CD, 2 μg); fluoroquinolones, including ciprofloxacin (CIP, 5 μg) and enrofloxacin (ENR, 5 μg); aminoglycosides, represented by gentamicin (CN, 10 μg); tetracyclines, represented by tetracycline (TE, 30 μg); phenicols, represented by chloramphenicol (C, 30 μg); ansamycins, represented by rifampicin (RD, 30 μg); fusidanes, represented by fusidic acid (FA, 10 μg); folate pathway inhibitors, represented by sulfamethoxazole–trimethoprim (SXT, 23.75/1.25 μg); and oxazolidinones, represented by linezolid (LNZ, 30 μg). Antimicrobial susceptibility results were interpreted following EUCAST clinical breakpoints. Enrofloxacin, a veterinary drug, was interpreted using criteria for animals by the Clinical and Laboratory Standards Institute [16]. Specifically, detection of methicillin resistance differed depending on the species and the antibiotic used: cefoxitin disk diffusion was employed as the reference method for the detection of methicillin resistance in *Staphylococcus aureus* and in coagulase-negative staphylococci. In contrast, for *Staphylococcus intermedius* and *S. pseudintermedius*, oxacillin was used as the primary marker for methicillin resistance, as cefoxitin is not a reliable surrogate for *mec*A-mediated resistance in this species according to EUCAST [14] recommendations. Molecular confirmation of methicillin resistance genes (*mec*A or *mec*C) was not performed; therefore, methicillin resistance was inferred exclusively based on phenotypic susceptibility testing according to EUCAST recommendations.

Additionally, isolates were categorized as multidrug-resistant (MDR), extensively drug-resistant (XDR), or pandrug-resistant (PDR) strains following the criteria outlined by Magiorakos et al. [15]. Specifically, strains were considered MDR if they were resistant to at least one antimicrobial agent in three or more antibiotic categories, XDR if they were resistant to all but one or two categories (i.e., remaining susceptible to only one or two categories) and PDR if they showed resistance to all tested agents across all antibiotic categories.

### 2.7. Biofilm Formation Assay by Crystal Violet Staining

The biofilm-forming capacity of *Staphylococcus* spp. isolates was evaluated using the quantitative crystal violet assay following the protocol described by Stepanovic et al. [17], with slight modifications as previously detailed by Nocera et al. [18]. Specifically, overnight cultures were adjusted to an OD_600_ of 0.2 (≈10^8^ CFU/mL) in fresh Brain Heart Infusion (BHI) broth (Liofilchem srl, Teramo, Italy). The standardized suspensions were then diluted at a ratio of 1:2 in 200 μL of BHI and transferred into sterile, flat-bottomed 96-well polystyrene microplates (Corning Inc., New York, NY, USA). Wells containing only 200 μL of BHI served as negative controls to confirm medium sterility and establish background absorbance levels. Microplates were incubated at 37 °C for 24 h, after which the contents were discarded and the wells were gently rinsed three times with sterile phosphate-buffered saline (PBS) to remove non-adherent cells, followed by air-drying. Biofilms attached to the well surfaces were stained with 200 μL of 1% crystal violet (BioMérieux, Marcy l’Etoile, France) for 30 min at room temperature. Excess stain was eliminated by washing with distilled water, and the retained dye was solubilized with 150 μL of 100% ethanol for 15 min at room temperature. Absorbance was measured at 570 nm using a Multiskan FC microplate reader (Thermo Fisher Scientific, Milan, Italy). All assays were performed in triplicate and repeated on three independent occasions.

Biofilm production was interpreted according to the criteria by Stepanovic et al. [17] by comparing each isolate’s mean OD with the negative control and the cutoff value (ODc). Based on these parameters, isolates were categorized as:

- No biofilm formation: OD ≤ ODc;
- Weak biofilm formation: ODc < OD ≤ 2 × ODc;
- Moderate biofilm formation: 2 × ODc < OD ≤ 4 × ODc;
- Strong biofilm formation: OD > 4 × ODc.

### 2.8. Data Analysis

The difference in the frequency of questionnaire answers, *Staphylococcus* spp. proportions between the two different Veterinary Teaching Hospitals, and the antibiotic resistance between the two VTHs and dogs and owners were investigated using Fisher’s exact test. For analyses involving multiple comparisons on the same samples, *p*-values were adjusted using the Benjamini–Hochberg procedure to control the false discovery rate. Logistic regression models were used to evaluate the association between questionnaire variables and the detection of shared *Staphylococcus* species in dog–owner pairs. Variables were initially screened through univariate analyses, and those with *p* < 0.20 were considered for inclusion in the multivariate model. To reduce the risk of overfitting, the number of predictors included in the final model was guided by the events-per-variable (EPV) criterion, ensuring an EPV value > 10. Collinearity among predictors was assessed using the variance inflation factor (VIF). Manual backward stepwise selection was applied to define the final model, which was guided by a combination of statistical and biological considerations. Changes in model deviance and likelihood ratio tests were used to compare nested models, ensuring that exclusion of variables improved model fit and that the results were consistent across alternative model specifications. Model selection was further supported by an evaluation of the information criteria (AIC). Model performance was evaluated by assessing the goodness of fit using the Hosmer–Lemeshow test; model discrimination was determined using the area under the receiver operating characteristic curve (AUC), and explanatory power was obtained using Nagelkerke’s pseudo-R^2^. A threshold of 0.05 was used to guide the interpretation of statistical differences. Statistical analyses were conducted in the R statistical environment (version 4.4.3) [19]. Logistic regression models were fitted using the package lme4 (version 1.1-36). Fisher’s exact tests performed within the Naples study group were calculated using the Easy Fisher Exact Test Calculator (www.socscistatistics.com accessed on 10 November 2025) web tool.

## 3. Results

### 3.1. Survey Data Collected from Dog Owners

More than 100 questionnaires were completed at each hospital, with a total of 120 questionnaires collected at VTH1 (Naples Province) and 160 at VTH2 (Turin Province). The response rates to individual questions are summarized in Table 1. In Naples Province, most animals lived exclusively indoors (56.7%), similar to those in Turin (50.0%). However, dogs living in Naples Province were more frequently confined to the garden compared to dogs from the Province of Turin (*p* = 0.023), which more frequently had access to both outdoor and indoor areas (*p* = 0.048). The dogs’ access to a personal resting place, including beds and sofas, was reported at comparable rates in Naples and Turin.

Regarding antiparasitic treatments, owners in the Province of Turin reported a lower overall frequency of administration. A higher proportion declared never using antiparasitic treatments, and flea and tick control was less frequently reported in Turin compared with the Province of Naples (*p* = 0.043). Also, constant and/or intermittent use was more common in Naples, whereas seasonal administration predominated in Turin (*p* < 0.001).

Regarding feeding habits, commercial diets were the most frequently reported in both Naples (54.2%) and Turin Provinces (61.9%), while homemade diets were relatively uncommon in both areas (12.5% and 10.0%, respectively).

Analysis of the collected data on close physical interactions showed that most owners in both Naples (75%) and Turin (80%) reported a close relationship with their pets, which was characterized by cuddling, hugging, and kissing. Hand hygiene practices after contact with pets were comparable between the two provinces, with “sometimes” being the most frequently reported response, followed by “always” and “never.” All-day interaction with pets was reported at similar rates in Turin (44.4%) and Naples (37.5%), while half-day interaction showed modest non-significant differences, being more common in Naples (49.2%) than in Turin (37.5%).

Regarding antibiotic therapy in pets, recent antibiotic use was similar between provinces, whereas dogs from the Province of Turin were more frequently reported as never having received antibiotics and less frequently as treated in the past (*p* < 0.001). Conversely, no differences were observed for the owners.

The final logistic regression model included owner gender, dog sex, and frequency of handwashing after contact with the dog. Male owners were associated with lower odds of sharing *Staphylococcus* spp. with their dogs (OR = 0.37, 95% CI: 0.16–0.82, *p* = 0.017). Neutered male dogs were associated with higher odds of shared colonization (OR = 4.23, 95% CI: 1.08–18.99, *p* = 0.045), although with limited precision. Similarly, owners who reported always washing their hands after contact with their dogs were associated with increased odds of sharing the same *Staphylococcus* species (OR = 10.33, 95% CI: 1.69–201.50, *p* = 0.035), although the wide confidence interval indicates substantial uncertainty in the magnitude of the effect.

Model performance was evaluated in terms of goodness of fit, discrimination, and explanatory power. The Hosmer–Lemeshow test did not indicate a lack of fit (*p* = 0.899), suggesting adequate agreement between the observed and predicted values. The model showed acceptable discrimination, with an area under the receiver operating characteristic curve (AUC) of 0.699. The explanatory power of the model was limited, as indicated by a Nagelkerke’s pseudo-R^2^ of 0.143. Considering the number of estimated parameters in the model, the events-per-variable (EPV) ratio was slightly below the conventional threshold of 10, indicating a potential risk of overfitting. This may have contributed to the limited precision and wide confidence intervals observed for some predictors.

### 3.2. Identification of Staphylococcus spp. and Species Distribution

A total of 477 *Staphylococcus* strains were recovered from nasal swabs of dogs and their owners attending the two Veterinary Teaching Hospitals (Table 2). Overall, *S. epidermidis* was the most frequently identified species (142/477; 29.8%), followed by *S. aureus* (106/477; 22.2%) and *S. pseudintermedius* (61/477; 12.8%). All other species were detected at lower frequencies (<7.0%).

Among dogs from Naples Province (VTH1; n = 73 isolates), *S. pseudintermedius* was the predominant species (26/73; 35.6%), followed by *S. aureus* (12/73; 16.4%) and *S. epidermidis* (7/73; 9.6%). Other species were detected at lower frequencies, including *S. warneri* (5.5%), *S. felis* (5.5%), and *S. simulans* (5.5%).

Similarly, in dogs from Turin Province (VTH2; n = 130 isolates), *S. pseudintermedius* was the most frequently isolated species (31/130; 23.8%), followed by *S. aureus* (21/130; 16.2%) and *S. intermedius* (15/130; 11.5%). As expected, owners showed a predominance of human-associated coagulase-negative staphylococci. Among owners from Naples Province (VTH1; n = 86 isolates), *S. epidermidis* was the most prevalent species (53/86; 61.6%), followed by *S. aureus* (27/86; 31.4%). No isolates of *S. pseudintermedius* were detected in this group.

Among owners from Turin Province (VTH2; n = 188 isolates), *S. epidermidis* remained the most frequent species (74/188; 39.4%), followed by *S. aureus* (46/188; 24.5%). Low percentages of *S. pseudintermedius* were detected in this group (4/188; 2.1%). Other species identified in owners included *S. warneri* (9.0%), *S. capitis* (8.5%), and *S. haemolyticus* (4.8%).

Overall, a clear host-related pattern emerged in both provinces: *S. pseudintermedius* predominated in dogs, whereas *S. epidermidis* was the dominant species in owners. The relative distribution of the two most clinically relevant species, *S. aureus* and *S. pseudintermedius*, was broadly comparable between Naples and Turin in dogs, while owners from Naples showed a higher proportion of *S. epidermidis* compared with those from Turin.

To investigate the potential sharing of *Staphylococcus* spp., species concordance was evaluated in matched dog–owner pairs from both study provinces.

Concordant species (isolation of the same *Staphylococcus* species in both dogs and owners) were identified in 54/280 dog–owner pairs (19.2%) overall. When subdivided by province, concordance was observed in 12/120 dog–owner pairs (10%) in Naples and in 42/160 dog–owner pairs (26.3%) in Turin Province (*p* = 0.001).

The remaining dog–owner pairs (226/280; 80.7%) showed discordant species profiles, defined as isolation of different *Staphylococcus* species in the dog and the corresponding owner.

Among concordant dog–owner pairs, the most frequently shared species was *S. aureus*, which was detected in 6/12 dog–owner pairs (50.0%) in the Province of Naples and 11/42 dog–owner pairs (26.2%) in the Province of Turin. *S. epidermidis* represented the second most common shared species, accounting for 5/12 pairs (41.7%) in Naples and 9/42 dog–owner pairs (21.4%) in Turin. *S. warneri* was also identified among concordant dog–owner pairs, with 1/12 dog–owner pairs (8.3%) in Naples and 7/42 dog–owner pairs (16.7%) in Turin. *S. pseudintermedius/intermedius* was identified as a shared species only in Turin Province (6/42 dog–owner pairs; 14.3%) overall. Other species were rarely involved in concordant colonization events and were detected only sporadically.

### 3.3. Antimicrobial Resistance Profiles

The antimicrobial resistance analysis was performed exclusively on the subset of 54 same-species dog–owner pairs (108 isolates) selected for pairwise comparison. Consequently, the resistance profiles reported here should be interpreted with caution and not as representative of the overall resistance prevalence among all isolates collected in the study. Resistance to at least one antimicrobial agent was detected in 94/108 isolates (87.0%) from the same-species dog–owner pairs. The proportion of resistant isolates was comparable between sites, accounting for 22/24 isolates (91.6%) in Naples and 72/84 isolates (85.7%) in Turin (*p* = 0.731).

**Figure 1:**
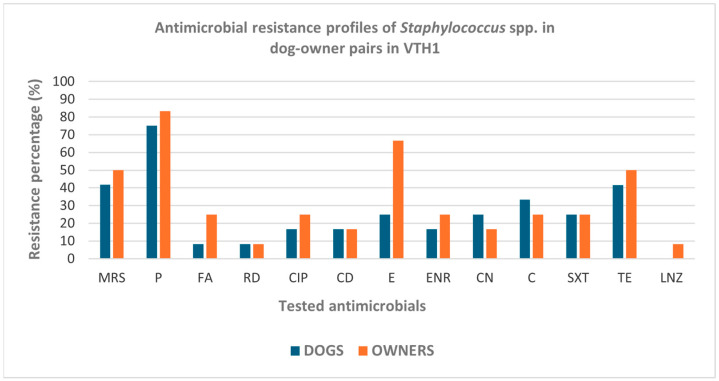
Antimicrobial resistance profiles of *Staphylococcus* spp. strains isolated from 12 dog–owner pairs in VTH1. MRS: methicillin-resistant staphylococci; P: penicillin; FA: fusidic acid; RD: rifampicin; CIP: ciprofloxacin; CD: clindamycin; E: erythromycin; ENR: enrofloxacin; CN: gentamicin; C: chloramphenicol; SXT: sulfamethoxazole–trimethoprim; TE: tetracycline; LNZ: linezolid.

In Naples, higher proportions of resistance to penicillin, fusidic acid, ciprofloxacin, clindamycin, enrofloxacin, erythromycin, and chloramphenicol, as well as a higher frequency of MRS strains, were observed in human-derived *Staphylococcus* spp. compared with canine isolates (Figure 1); however, these differences were not statistically significant.

**Figure 2:**
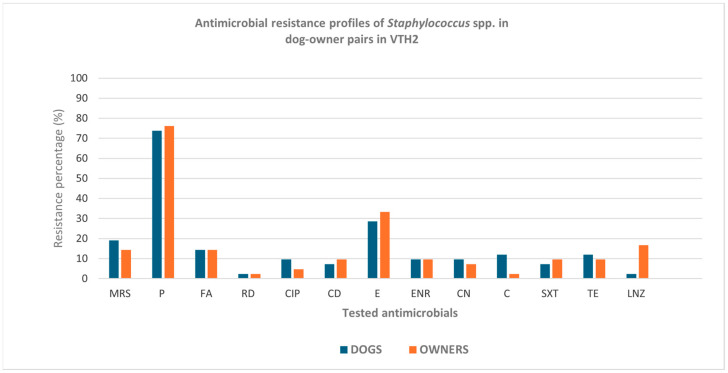
Antimicrobial resistance profiles of *Staphylococcus* spp. strains isolated from 42 dog–owner pairs in VTH2. MRS: methicillin-resistant staphylococci; P: penicillin; FA: fusidic acid; RD: rifampicin; CIP: ciprofloxacin; CD: clindamycin; E: erythromycin; ENR: enrofloxacin; CN: gentamicin; C: chloramphenicol; SXT: sulfamethoxazole–trimethoprim; TE: tetracycline; LNZ: linezolid.

Similarly, in Turin, higher resistance percentages in human-origin isolates were observed for penicillin, clindamycin, erythromycin, sulfamethoxazole–trimethoprim, and linezolid, as well as a higher proportion of MRS compared with those of canine origin. Also in this case, the differences were not statistically significant (Figure 2).

Overall, multidrug resistance (MDR) was observed in 37/108 isolates (34.3%). In Naples Province, MDR was detected in 5/12 (41.7%) dogs and 8/12 (66.7%) owners. MDR was more common in Naples than Turin (13/24, 54.2% vs. 24/84, 28.6%), and within each site, it was detected more frequently among owner isolates than dog isolates (Naples: 8/12 vs. 5/12; Turin: 13/42 vs. 11/42). The difference between provinces was statistically significant (*p* = 0.028), but no statistical significance was observed for different MDR categories between dogs and owners. No XDR or PDR phenotypes were detected among the isolates analyzed.

### 3.4. Methicillin Resistance Patterns

Methicillin resistance was assessed phenotypically according to EUCAST (2025) recommendations, using oxacillin or cefoxitin disk diffusion, with positive results of both tests indicative of penicillin-binding protein 2a (PBP2a)-mediated resistance. Resistant isolates were detected in both study areas, although with different prevalence rates and patterns of host distribution.

In the Province of Naples (Table 3a), methicillin resistance was detected in 11 of 24 isolates (45.8%). Considering host origin, resistant isolates were identified in 5 of 12 dogs (41.7%) and 6 of 12 owners (50%) (Table 3a, Figure 1). Concordant methicillin resistance profiles were observed in seven pairs (58.3%), while five pairs (41.7%) showed discordant profiles.

In the Province of Turin (Table 3b), methicillin resistance was detected in 14 of 84 isolates (16.7%) and occurred in 8 of 42 (14.3%) owner isolates and 6 of 42 (19%) dog isolates (Table 3b and Figure 2). A high level of concordance was observed, with 38 of 42 dog–owner pairs (90.5%) showing concordant methicillin resistance phenotypes and only four dog–owner pairs (9.5%) displaying discordance.

Overall, these findings indicate a higher prevalence of phenotypically methicillin-resistant staphylococci in Naples than in Turin (*p* = 0.035) and highlight differences in host distribution and species concordance in dog–owner pairs between the two study sites.

When considering resistance markers at the couple level in both provinces, penicillin resistance, used as a phenotypic indicator of penicillinase production, was prevalent, being detected in 32/54 dog–owner pairs (59.3%). In contrast, resistance to oxacillin or cefoxitin, reliable phenotypic predictors of methicillin resistance, was less frequently observed (9/54, 16.7%). Specifically, methicillin resistance profiles were identified in 4 of 12 dog–owner pairs (33.3%) in Naples, whereas in Turin, such profiles were detected in 5 of 42 dog–owner pairs (11.9%).

Furthermore, dog–owner pairs in which both members carried multidrug-resistant (MDR) staphylococci were detected in 3 out of 12 pairs (25%) in Naples, and in 7 out of 42 dog–owner pairs (16.7%) in Turin, showing no statistically significant difference. In addition, identical phenotypic profiles, defined by the same penicillin, oxacillin or cefoxitin resistance pattern, MDR status, and biofilm production type, were observed in 4 out of 12 dog–owner pairs (33.3%) in Naples and in 3 out of 42 dog–owner pairs (7.1%) in Turin, representing a statistically significant difference (*p* = 0.002) (Table 3a,b and Table S1a,b). In Naples, when phenotypes were aggregated by host (Table 3a), penicillinase production was more frequently observed among owners (10/12, 83.3%) than dogs (9/12, 75%). Equally, methicillin resistance showed a slightly higher frequency in owners (6/12, 50%) than in dogs (5/12, 41,7%). Multidrug resistance was more common in owners (8/12, 66.7%) compared to dogs (5/12, 41.7%). In Turin (Table 3b), penicillinase production was observed with comparable frequencies in dogs (31/42, 73.8%) and owners (32/42, 76.2%). Similarly, methicillin resistance showed only marginal differences between the two groups, being detected in 6/42 dogs (14.3%) and 8/42 owners (19.0%). Multidrug resistance was higher in owners (13/42, 31%) than in dogs (11/42, 26.2%), although the overall prevalence remained moderate in both populations.

### 3.5. Biofilm Formation Ability

**Figure 3:**
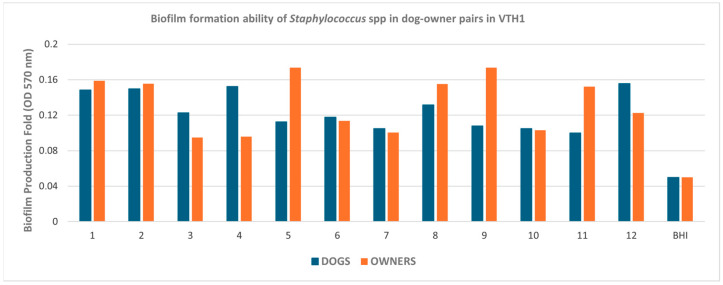
Biofilm-forming ability of *Staphylococcus* spp. strains isolated from 12 dog–owner pairs in Naples. Biofilm production ability was determined using the mean OD 570 nm of the negative control (0.05), represented by Brain Heart Infusion (BHI) broth. Values < 0.05 were considered non-biofilm producers; values between 0.05 and 0.1 (2× the negative control value of 0.05) were considered weak producers; values between 0.1 and 0.2 (4× the negative control value of 0.05) were considered moderate producers; values > 0.2 (>4× the negative control value of 0.05) were considered strong producers.

**Figure 4:**
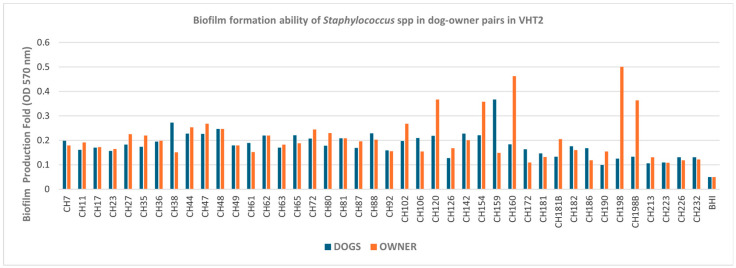
Biofilm-forming ability of *Staphylococcus* spp. strains isolated from 42 dog–owner pairs in Turin. Biofilm production ability was determined using the mean OD 570 nm of the negative control (0.05), represented by Brain Heart Infusion (BHI) broth. Values < 0.05 were considered non-biofilm producers; values between 0.05 and 0.1 (2× the negative control value of 0.05) were considered weak producers; values between 0.1 and 0.2 (4× the negative control value of 0.05) were considered moderate producers; values > 0.2 (>4× the negative control value of 0.05) were considered strong producers.

The assessment of biofilm-forming capacity demonstrated that, in Naples, only two dog–owner pairs exhibited discordant biofilm production. In these cases, *S. epidermidis* isolates from dogs and their respective owners were classified as moderate biofilm producers when they were of canine origin and as weak producers when they were of human origin (Figure 3). In Turin, 24 out of 42 pairs displayed concordant biofilm-forming capacity (Figure 4).

In Naples, most of the isolates related to the selected dog–owner pairs, specifically 22 out of 24, were moderate biofilm producers, while the remaining two strains were weak biofilm producers (Table 3a). In any case, we found no association between biofilm-forming ability and MDR profiles (*p* = 0.482): among isolates with moderate biofilm-forming ability (n = 22), 11 were MDR, while 11 were non-MDR.

In Turin, we detected 33 strong biofilm-producing strains, 11 of which also showed an MDR profile (Table 3b). The two groups did not differ significantly, indicating no correlation between strong biofilm-forming ability and MDR profiles (*p* = 1.000).

## 4. Discussion

The present study investigated the distribution, antimicrobial resistance profiles, and biofilm-forming ability of *Staphylococcus* spp. isolated from dog–owner pairs in two geographically distinct Italian areas, with a specific focus on species concordance.

The genus *Staphylococcus* comprises more than 50 species and 24 subspecies and is known for its ability to survive a broad range of adverse environmental conditions, including extreme temperatures, dehydration, and low water activity [20,21]. Within the “One Health” framework, staphylococci are of particular concern because they colonize both humans and animals and can act as reservoirs for antimicrobial resistance genes with potential for interspecies transmission [22].

Overall, the species distribution observed in the present study was consistent with the expected host-specific ecological niches of staphylococci. In both geographical areas, *S. pseudintermedius* was the predominant species isolated from dogs, confirming its well-recognized role as the primary canine commensal and opportunistic pathogen [23]. Conversely, among owners, coagulase-negative staphylococci, particularly *S. epidermidis*, were highly prevalent, especially in Naples, while both *S. epidermidis* and *S. aureus* were frequently detected in Turin. This distribution is consistent with previous reports indicating that *S. epidermidis* represents one of the most common colonizers of the human nasal mucosa [24], whereas *S. aureus* maintains a stable carriage rate in a substantial proportion of healthy individuals [25]. Both *S. aureus* and *S. epidermidis* are especially significant due to their involvement in infections ranging from mild skin lesions to severe, invasive diseases such as bacteremia, endocarditis, and device-associated infections [26,27]. In addition, *S. aureus* and *S. epidermidis* represented the most frequently shared species between dogs and owners in concordant pairs, with no significant difference observed between the two study populations. The frequent detection of *S. aureus* and *S. epidermidis* in concordant dog–owner pairs may reflect shared exposure or co-colonization within the household environment rather than direct transmission, particularly because strain-level typing was not performed in the present study. Previous research has shown host-specific colonization preferences and a higher adhesion capacity of *S. aureus* to human epithelial cells [28,29], which may partly explain the predominance of human-associated species observed among owners. However, the cross-sectional design of this study does not allow conclusions regarding the directionality or mechanisms underlying the observed species concordance. Studies using molecular typing approaches have demonstrated that genetically related staphylococcal strains can occasionally be identified in dogs and their owners living in the same household [28,30], supporting the possibility of bacterial sharing or exposure to common sources. Similarly, epidemiological investigations conducted in Italy have reported overlapping *S. aureus* and *S. epidermidis* strains in dogs and their owners, suggesting that close human–animal contact may facilitate microbial exchange within domestic environments [7]. Nevertheless, confirmation of strain-level relatedness would require molecular or genomic analyses, which were beyond the scope of the present study.

Although differences in pet management practices were observed between the Naples and Turin populations, these variables were not associated with the occurrence of species concordance within dog–owner pairs. The associations observed between potential sharing and owner gender, dog sex, and handwashing practices should be interpreted with caution. While male owners appeared less likely to share *Staphylococcus* spp. with their dogs, gender-related behavioral differences in daily pet interaction patterns (e.g., close contact, caregiving role, and duration of physical interaction) may partially explain this finding. The associations observed for neutered male dogs and for handwashing practices are difficult to interpret and may reflect unmeasured confounding or behavioral patterns not captured by the questionnaire. Importantly, the wide confidence intervals, particularly for the handwashing variable (95% CI: 1.69–201.50), indicate limited precision of the estimated effects and suggest a degree of model uncertainty, likely related to the relatively small number of observations in some exposure categories. In addition, the overall explanatory power of the model was modest, reflecting the multifactorial nature of the investigated outcome. Taken together, these findings should be considered exploratory and hypothesis-generating rather than indicative of causal relationship.

The absence of differences in antimicrobial resistance profiles between canine and human isolates within shared species is consistent with potential strain sharing, although molecular confirmation at strain-level was beyond the scope of this study. In contrast, some differences were observed between the two geographical areas, namely higher resistance to chloramphenicol and tetracycline and an increased frequency of phenotypically methicillin-resistant isolates detected in Naples. While the lack of molecular screening for *mec*A and *mec*C genes for the characterization of methicillin resistance mechanisms represents a limitation of this study, these phenotypic findings may reflect regional variability in antimicrobial exposure or the circulation of local strains rather than host-specific selective pressures. MDR isolates were detected in both geographical areas, with no significant differences in their frequency between locations. The overall MDR proportion remained moderate, supporting the hypothesis that community-associated staphylococcal populations are not yet characterized by widespread high-level resistance. In our study, no isolates fulfilled the criteria for the XDR or PDR phenotypes, indicating that, despite the detection of multidrug resistance, extreme resistance profiles remain uncommon in this community-based population.

A key element of staphylococci’s persistence and pathogenicity is the biofilm-forming ability [31], which greatly enhances bacterial survival under hostile conditions, including exposure to antimicrobial agents and host immune defenses [32], and leads to higher rates of treatment failure and frequent recurrence [33,34]. Evidence suggests that biofilm producers often exhibit elevated antimicrobial resistance, either through specific genetic determinants or through the intrinsic protective properties of the biofilm matrix [35,36]. Specifically, MRSA often displays enhanced biofilm-forming capacity, which further complicates therapeutic management [37,38]. Understanding these traits is essential when evaluating the potential sharing of *Staphylococcus* spp. between dogs and their owners, as biofilm-associated and resistant strains may be more capable of persisting and disseminating across species barriers. However, in this study, no association was detected between biofilm production and multidrug resistance in either study population. While these results should be interpreted with caution due to the limited number of isolates available for this specific analysis, these findings are consistent with several previous studies reporting no clear relationship between biofilm-forming ability and antimicrobial resistance in *Staphylococcus* populations [39,40,41] and support the theory that the relationship between biofilm biomass and antimicrobial susceptibility is highly variable and is influenced by species, strain-specific traits, type of antibiotic, and methodological approaches [42]. Another plausible explanation is the predominance of commensal rather than clinical isolates in this study; the isolates we examined likely represent saprophytic populations of the nasal mucosa in both dogs and humans, which may experience a different evolutionary drive to develop multidrug resistance or enhanced biofilm formation.

Several limitations should be considered when interpreting these findings. The cross-sectional design of this study prevents assessment of temporal dynamics or the directionality of bacterial sharing between hosts. Although bacterial identification was performed at the species level using MALDI-TOF MS, the absence of molecular typing methods, such as MLST, spa typing, or whole-genome sequencing, precluded determination of strain-level relatedness between isolates. Similarly, methicillin resistance was inferred from phenotypic profiles without molecular confirmation of *mec* genes. It should also be noted that evaluation of antimicrobial resistance was restricted to isolates from concordant dog–owner pairs to better investigate phenotypic indicators of sharing. While this focus addressed this study’s primary objective, it introduced selection bias; consequently, the reported resistance frequencies may not reflect the broader staphylococcal population but are instead specifically representative of isolates involved in species-level concordance. Regarding the study population, recruitment of dogs from referral Veterinary Teaching Hospitals and reliance on convenience sampling may limit the generalizability of the findings to broader canine and human populations. While the sample size may appear modest, it reflects the practical challenges of recruiting dog–owner pairs for nasal swab collection, a procedure often perceived as uncomfortable, particularly in the post-pandemic context. Consequently, the reported frequencies should not be interpreted as prevalence estimates or assumed to be fully representative of the broader population.

A comprehensive One Health investigation would ideally include environmental sampling; however, domestic environments shared by dogs and owners are highly heterogeneous, making standardized and reproducible sampling logistically challenging. Finally, the lack of molecular or genomic typing prevents confirmation of species concordance at the strain level between dogs and owners. Future studies incorporating molecular or genomic approaches will be essential to better characterize the transmission dynamics suggested by these findings.

## 5. Conclusions

The identification of shared *Staphylococcus* spp., particularly *S. aureus* and *S. epidermidis*, in dogs and their owners supports the occurrence of species concordance and potential microbial sharing within households. The absence of differences in antimicrobial resistance profiles between human- and animal-origin isolates, together with the predominance of human-associated species, is consistent with the presence of a shared ecological niche of commensal staphylococci at the human–dog interface. In addition, no significant association emerged between biofilm-forming ability and multidrug resistance. Overall, these findings highlight the complexity of staphylococcal ecology within households and suggest that microbial sharing between dogs and owners may reflect stable co-colonization dynamics rather than simple transmission events. A more comprehensive One Health approach, potentially including environmental sampling and strain-level characterization, would be necessary to better understand these interactions.

## Figures and Tables

**Table 1:** Demographic, lifestyle, and clinical characteristics of dog–owner pairs from two Italian Veterinary Teaching Hospitals (VTHs).

Variable	Category	VTH1 n. (%)	VTH2 n. (%)	Fisher’s Test *p*	Adjusted *p*
**SECTION A: LIFESTYLE AND MANAGEMENT**					
**Living** **environment**	home	68 (56.7%)	80 (50.0%)	0.279	0.470
	garden	12 (10.0%)	3 (1.9%)	0.005	**0.023**
	both	40 (33.3%)	77 (48.1%)	0.015	**0.048**
**Daily habits**	personal resting place	10 (8.3%)	13 (8.1%)	1.000	1.000
	access to bed and sofa	48 (40.0%)	45 (28.1%)	0.041	0.109
	resting place, bed, sofa	62 (51.7%)	102 (63.4%)	0.050	0.123
**Parasite Treatment**	No administration	0 (0.0%)	9 (5.8%)	0.012	**0.043**
	flea and tick control	130 (87.8%)	97 (63.0%)	0.012	**0.043**
	with repellents	30 (20.2%)	47 (30.5%)	0.499	0.694
	only repellents	0 (0.0%)	1 (0.7%)	1.000	1.000
	constant use	102 (68.9%)	33 (23.2%)	<0.001	**<0.001**
	intermittent use	22 (14.9%)	2 (1.4%)	<0.001	**<0.001**
	monthly use	26 (17.6%)	11 (7.7%)	0.001	**0.003**
	seasonal use	21 (14.1%)	96 (67.6%)	<0.001	**<0.001**
**Feeding habits**	homemade diet	15 (12.5%)	16 (10.0%)	0.566	0.755
	commercial diet	65 (54.2%)	99 (61.9%)	0.228	0.429
	mixed diet	40 (33.3%)	45 (28.1%)	0.361	0.550
**SECTION B: PET-OWNER INTERACTION AND HYGIENE PRACTICES**					
**Pet-owner** **interaction**	cuddles	10 (8.3%)	14 (8.8%)	1.000	1.000
	cuddles, hugs	20 (16.7%)	18 (11.2%)	0.219	0.429
	cuddles, hugs, kisses	90 (75.0%)	128 (80.0%)	0.383	0.527
**Hand washing** **after pet contact**	always	23 (19.2%)	33 (20.6%)	0.880	1.000
	sometimes	79 (65.8%)	103 (64.4%)	0.899	1.000
	never	18 (15.0%)	24 (15.0%)	1.000	1.000
**Interaction time** **with pets**	all day	45 (37.5%)	71 (44.4%)	0.271	0.470
	half day	59 (49.2%)	60 (37.5%)	0.067	0.153
	evening only	16 (13.3%)	29 (18.1%)	0.326	0.522
**SECTION C: ANTIBIOTIC USE HISTORY**					
**Antibiotic therapy** **in pets**	never	0 (0.0%)	26 (16.2%)	<0.001	**<0.001**
	recent, e.g., last month	36 (30.0%)	63 (39.4%)	0.130	0.277
	past, e.g., last year	84 (70.0%)	71 (44.4%)	<0.001	**<0.001**
**Antibiotic therapy** **in owners**	never	0 (0.0%)	6 (3.8%)	0.039	0.109
	recent, e.g., last month	25 (20.8%)	29 (18.1%)	0.647	0.828
	past, e.g., last year	95 (79.2%)	125 (78.1%)	0.884	1.000

Significant *p*-values (*p* < 0.05; *p* < 0.001) are highlighted in bold.

**Table 2:** Distribution of *Staphylococcus* species isolated from nasal swabs of dogs and their owners at two Italian Veterinary Teaching Hospitals (VTHs).

*Staphylococcus* Species	Total n. (%)	VTH1 Dogs n. (%)	VTH2 Dogs n. (%)	VTH1 Owners n. (%)	VTH2 Owners n. (%)
*S. epidermidis*	142 (29.8)	7 (9.6)	8 (6.2)	53 (61.6)	74 (39.4)
*S. aureus*	106 (22.2)	12 (16.4)	21 (16.2)	27 (31.4)	46 (24.5)
*S. pseudintermedius*	61 (12.8)	26 (35.6)	31 (23.8)	0 (0.0)	4 (2.1)
*S. capitis*	33 (6.9)	3 (4.1)	14 (10.8)	0 (0.0)	16 (8.5)
*S. warneri*	30 (6.3)	4 (5.5)	7 (5.4)	2 (2.3)	17 (9.0)
*S. intermedius*	22 (4.6)	2 (2.7)	15 (11.5)	0 (0.0)	5 (2.7)
*S. haemolyticus*	18 (3.8)	3 (4.1)	6 (4.6)	0 (0.0)	9 (4.8)
*S. hominis*	11 (2.3)	1 (1.4)	7 (5.4)	1 (1.2)	2 (1.1)
*S. xylosus*	10 (2.1)	2 (2.7)	5 (3.8)	0 (0.0)	3 (1.6)
*S. lugdunensis*	9 (1.9)	0 (0.0)	0 (0.0)	2 (2.3)	7 (3.7)
*S. felis*	5 (1.0)	4 (5.5)	1 (0.8)	0 (0.0)	0 (0.0)
*S. simulans*	5 (1.0)	4 (5.5)	0 (0.0)	1 (1.2)	0 (0.0)
*S. saprophyticus*	5 (1.0)	0 (0.0)	2 (1.5)	0 (0.0)	3 (1.6)
Other species ^§^	20 (4.2)	5 (6.8)	13 (10.0)	0 (0.0)	2 (1.1)
Total	477	73	130	86	188

^§^ Other species include: *S. equorum*, *S. nepalensis*, *S. lentus*, *S. schleiferi*, *S. coagulans*, *S. caprae*, *S. cohnii*, *S. condimenti*, *S. pasteuri*, *S. sciuri*, and *S. ureolyticus* (each ≤ 1.0% per group).

**Table 3:** (**a**) Phenotypic characteristics of *Staphylococcus* isolates recovered from 12 dog–owner pairs in Naples (n = 24 isolates). (**b**) Phenotypic characteristics of *Staphylococcus* isolates recovered from 42 dog–owner pairs in Turin (n = 84 isolates).

(**a**)				
**Phenotype**	**Dogs n. (%)**	**Owners n. (%)**	**Fisher’s Exact *p***	**Adjusted *p***
**Penicillinase production**	9/12 (75%)	10/12 (83.3%)	1.000	1.000
**Methicillin resistance (MRS)**	5/12 (41.7%)	6/12 (50%)	1.000	1.000
**Multidrug resistance (MDR)**	5/12 (41.7%)	8/12 (66.7%)	0.414	0.797
**Biofilm-forming ability:**				
Weak	0	2/12 (16.7%)	0.478	0.797
Moderate	12/12 (100%)	10/12 (83.3%)	0.478	0.797
Strong	0	0	-	
Non-producer	0	0	-	
(**b**)				
**Phenotype**	**Dogs n. (%)**	**Owners n. (%)**	**Fisher’s exact *p***	**Adjusted *p***
**Penicillinase production**	31/42 (73.8%)	32/42 (76.2%)	1.000	1.000
**Methicillin resistance (MRS)**	6/42 (14.3%)	8/42 (19%)	0.771	1.000
**Multidrug resistance (MDR)**	11/42 (26.2%)	13/42 (31%)	0.810	1.000
**Biofilm-forming ability:**				
Weak	1/42 (2.4%)	0	1.000	1.000
Moderate	27/42 (64.3%)	23/42 (54.8%)	0.505	1.000
Strong	14/42 (33.3%)	19/42 (45.2%)	0.372	1.000
Non-producer	0	0		

## Data Availability

The data supporting the findings of this study are available within this article.

## Supplementary Materials

The following supporting information can be downloaded at: https://www.mdpi.com/article/10.3390/pathogens15040356/s1, Table S1: (a) Phenotypic indicators of penicillinase production, *mec*A-mediated methicillin resistance, multidrug resistance (MDR), and biofilm-forming ability among paired *Staphylococcus* isolates from dogs and owners in the Province of Naples (Southern Italy). (b) Phenotypic indicators of penicillinase production, *mec*A-mediated methicillin resistance, and multidrug resistance (MDR) among paired *Staphylococcus* isolates from dogs and owners (Turin Province, Northern Italy).
