# Supplementary material for: Investigating the Sharing of Staphylococcus spp. Between Dogs and Their Owners: A Comparative Study from Two Italian Veterinary Teaching Hospitals
Source: Pathogens. 2026 Mar 27;15(4):356. doi: 10.3390/pathogens15040356 (PMC13118325; doi:10.3390/pathogens15040356)
Supplement: Supplementary file 1 [file pathogens-15-00356-s001.zip › pathogens-4193904-supplementary.pdf]

Supplementary Materials

Table S1a. Phenotypic indicators of penicillinase production and *mecA*-mediated methicillin resistance, Multidrug Resistance (MDR), and biofilm-forming ability among paired *Staphylococcus* isolates from dogs and owners in the Province of Naples (Southern Italy).

Pair No.	Species	Origin	Penicillinase Production phenotype	Methicillin resistance (MRS) phenotype	Multidrug resistance (MDR) phenotype	Biofilm-forming ability
1	*S. aureus*	Dog	yes	yes	yes	Moderate
	S. aureus	Owner	yes	yes	yes	Moderate
2	*S. epidermidis*	Dog	yes	yes	yes	Moderate
	S. epidermidis	Owner	yes	yes	yes	Moderate
3	*S. epidermidis*	Dog	yes	no	no	Moderate
	S. epidermidis	Owner	yes	no	yes	Weak
4	*S. epidermidis*	Dog	no	no	no	Moderate
	S. epidermidis	Owner	yes	no	yes	Weak
5	*S. aureus*	Dog	yes	no	yes	Moderate
	S. aureus	Owner	yes	no	no	Moderate
6	*S. aureus*	Dog	yes	yes	yes	Moderate
	S. aureus	Owner	yes	yes	yes	Moderate
7	*S. aureus*	Dog	No	no	no	Moderate
	S. aureus	Owner	yes	no	no	Moderate
8	*S. warneri*	Dog	yes	no	no	Moderate
	S. warneri	Owner	yes	yes	yes	Moderate
9	*S. aureus*	Dog	yes	no	no	Moderate
	S. aureus	Owner	no	no	no	Moderate
10	*S. aureus*	Dog	yes	yes	no	Moderate
	S. aureus	Owner	yes	no	yes	Moderate
11	*S. epidermidis*	Dog	No	No	no	Moderate
	S. epidermidis	Owner	No	yes	no	Moderate
12	*S. epidermidis*	Dog	yes	yes	yes	Moderate
	S. epidermidis	Owner	yes	yes	yes	Moderate

Resistance to penicillin was interpreted as a phenotypic indicator of penicillinase production. *Mec*A-mediated methicillin resistance was assessed using oxacillin disk diffusion for coagulase-negative staphylococci and cefoxitin disk diffusion as the reference screening method for *S. aureus*, according to EUCAST guidelines.

Table S1b. Phenotypic indicators of penicillinase production and *mec*A-mediated methicillin resistance, Multidrug Resistance (MDR) among paired *Staphylococcus* isolates from dogs and owners (Turin Province, Northern Italy).

Pair No.	Species	Origin	Penicillinase Production phenotype	Methicillin resistance (MRS) phenotype	Multidrug resistance (MDR) phenotype	Biofilm-forming ability
CH7	*S. aureus*	Dog	yes	yes	yes	Moderate
	S. aureus	Owner	yes	yes	yes	Moderate
CH11	*S. aureus*	Dog	no	no	no	Moderate
	S. aureus	Owner	yes	no	no	Moderate
CH17	*S. aureus*	Dog	yes	no	no	Moderate

	S. aureus	Owner	yes	no	yes	Moderate
CH27	*S. aureus*	Dog	no	no	no	Moderate
	S. aureus	Owner	yes	no	no	Strong
CH36	*S. aureus*	Dog	no	no	no	Strong
	S. aureus	Owner	no	no	no	Moderate
CH44	*S. aureus*	Dog	no	no	no	Strong
	S. aureus	Owner	no	no	no	Strong
CH48	*S. aureus*	Dog	yes	no	no	Strong
	S. aureus	Owner	yes	no	no	Strong
CH61	*S. aureus*	Dog	yes	no	no	Moderate
	S. aureus	Owner	yes	no	no	Moderate
CH72	*S. aureus*	Dog	yes	no	yes	Strong
	S. aureus	Owner	yes	no	yes	Strong
CH88	*S. aureus*	Dog	yes	no	no	Strong
	S. aureus	Owner	yes	no	no	Strong
CH181	*S. aureus*	Dog	no	no	no	Moderate
	S. aureus	Owner	yes	no	no	Moderate
CH65	*S. capitis*	Dog	yes	no	no	Strong
	S. capitis	Owner	yes	no	no	Moderate
CH172	*S. capitis*	Dog	yes	no	yes	Moderate
	S. capitis	Owner	no	no	no	Strong
CH181B	*S. capitis*	Dog	yes	no	no	Moderate
	S. capitis	Owner	no	no	no	Moderate
CH186	*S. capitis*	Dog	no	no	no	Moderate
	S. capitis	Owner	yes	no	no	Strong
CH223	*S. capitis*	Dog	yes	no	no	Moderate
	S. capitis	Owner	no	no	no	Moderate
CH102	*S. capitis*	Dog	yes	no	yes	Moderate
	S. capitis	Owner	yes	no	no	Strong
CH23	*S. epidermidis*	Dog	yes	yes	yes	Moderate
	S. epidermidis	Owner	yes	yes	yes	moderate
CH62	*S. epidermidis*	Dog	yes	yes	yes	Strong
	S. epidermidis	Owner	yes	no	no	Strong
CH81	*S. epidermidis*	Dog	yes	no	no	Strong
	S. epidermidis	Owner	yes	no	no	Strong
CH87	*S. epidermidis*	Dog	yes	no	no	Moderate
	S. epidermidis	Owner	no	no	no	Moderate
CH120	*S. epidermidis*	Dog	yes	no	no	Moderate
	S. epidermidis	Owner	yes	no	no	Strong
CH142	*S. epidermidis*	Dog	yes	no	no	Strong
	S. epidermidis	Owner	yes	no	yes	Strong
CH190	*S. epidermidis*	Dog	yes	no	no	Weak
	S. epidermidis	Owner	yes	no	no	Moderate
CH213	*S. epidermidis*	Dog	no	no	no	Moderate
	S. epidermidis	Owner	yes	no	no	Moderate
CH198B	*S. epidermidis*	Dog	yes	no	no	Moderate
	S. epidermidis	Owner	yes	yes	yes	strong
CH49	*S. haemolyticus*	Dog	yes	yes	yes	Moderate

	S. haemolyticus	Owner	yes	yes	yes	Moderate
CH63	*S. intermedius*	Dog	yes	no	no	Moderate
	S. intermedius	Owner	yes	no	no	Moderate
CH106	*S. intermedius*	Dog	yes	no	yes	Strong
	S. intermedius	Owner	yes	no	yes	Moderate
CH198	*S. lugdunensis*	Dog	no	no	no	Moderate
	S. lugdunensis	Owner	yes	yes	yes	Strong
CH35	*S. pseudintermedius*	Dog	yes	no	no	Moderate
	S. pseudintermedius	Owner	no	no	no	Strong
CH38	*S. pseudintermedius*	Dog	yes	yes	yes	Strong
	S. pseudintermedius	Owner	yes	yes	yes	Moderate
CH80	*S. pseudintermedius*	Dog	yes	yes	yes	Moderate
	S. pseudintermedius	Owner	yes	yes	no	Strong
CH154	*S. pseudintermedius*	Dog	yes	no	yes	Strong
	S. pseudintermedius	Owner	yes	no	yes	Strong
CH92	*S. warneri*	Dog	no	no	no	Moderate
	S. warneri	Owner	no	no	no	Moderate
CH126	*S. warneri*	Dog	yes	no	no	Moderate
	S. warneri	Owner	yes	no	no	Moderate
CH159	*S. warneri*	Dog	no	no	no	Strong
	S. warneri	Owner	yes	no	yes	Moderate
CH160	*S. warneri*	Dog	yes	no	no	Moderate
	S. warneri	Owner	yes	yes	yes	Strong
CH182	*S. warneri*	Dog	no	no	no	Moderate
	S. warneri	Owner	no	no	no	Moderate
CH226	*S. warneri*	Dog	yes	no	no	Moderate
	S. warneri	Owner	no	no	no	Moderate
CH232	*S. warneri*	Dog	yes	no	no	Moderate
	S. warneri	Owner	yes	no	no	Moderate
CH47	*S. xylosus*	Dog	yes	no	no	Strong
	S. xylosus	Owner	yes	no	no	Strong

Resistance to penicillin was interpreted as a phenotypic indicator of penicillinase production. *Mec*A-mediated methicillin resistance was assessed using oxacillin disk diffusion for coagulase-negative staphylococci and cefoxitin disk diffusion as the reference screening method for *S. aureus*, according to EUCAST guidelines.
